# Ginsenoside Rg1 Alleviates Blood–Milk Barrier Disruption in Subclinical Bovine Mastitis by Regulating Oxidative Stress-Induced Excessive Autophagy

**DOI:** 10.3390/antiox13121446

**Published:** 2024-11-24

**Authors:** Shanshan Yang, Zihao Fang, Hongwei Duan, Weitao Dong, Longfei Xiao

**Affiliations:** 1 College of Veterinary Medicine, Gansu Agricultural University, Lanzhou 730070, China; yss1209@sina.com (S.Y.); fangzh210@163.com (Z.F.); grand6138@163.com (H.D.); 2 Animal Science and Technology College, Beijing University of Agriculture, Beijing 100096, China

**Keywords:** subclinical bovine mastitis, reactive oxygen species, autophagy, NLRP3 inflammasome, blood–milk barrier, ginsenoside Rg1

## Abstract

As a critical disease usually infected by *Staphylococcus aureus*, with a worldwide effect on dairy animals, subclinical mastitis is characterized by persistence and treatment resistance. During mastitis, the blood–milk barrier (BMB)’s integrity is impaired, resulting in pathogen invasion and milk quality decline. In this study, it was found that ginsenoside Rg1 (Rg1), a natural anti-inflammatory and antioxidant compound derived from ginseng, inhibited the onset of tight junction (TJ) dysfunction and ameliorated lipoteichoic acid (LTA)-induced BMB disruption inside and outside the organisms. According to subsequent mechanistic studies, Rg1 inhibited excessive autophagy and inactivated the NLRP3 inflammasome by blockading ROS generation, thereby alleviating TJ dysfunction. Peroxisome proliferator-activated receptor gamma (PPARγ) was identified as a potential target of Rg1 by means of molecular docking plus network pharmacology analysis. Furthermore, it was demonstrated that Rg1 inhibited the oxidative stress levels by activating PPARγ, and regulating the upstream autophagy-related AMPK/mTOR signaling pathway, thus decreasing excessive in vivo and in vitro autophagy. The ROS/autophagy/NLRP3 inflammasome axis was identified as a promising target for treating subclinical bovine mastitis in this study. In conclusion, Rg1 is proven to alleviate BMB disruption by activating PPARγ to inhibit oxidative stress and subsequent excessive autophagy in the case of subclinical bovine mastitis.

## 1. Introduction

Mastitis belongs to the group of inflammatory diseases with the highest incidence and prevalence rates in dairy cows, which can reduce the yield and quality of milk, resulting in substantial economic loss [1]. It can be classified as clinical mastitis and recessive mastitis. Lipoteichoic acid (LTA) is a component of the cell wall of *Staphylococcus aureus* (*S. aureus*), the most ubiquitous pathogen of mastitis, of which the mild but persistent pro-inflammatory activity is the main cause of subclinical chronic mastitis, which is very hard to detect and cure [2,3]. As a crucial biological barrier of the mammary gland, the blood–milk barrier (BMB) possesses the pivotal biological function of maintaining steady-state microenvironment regulation in mammary tissue, resisting pathogen invasion, and preventing the loss of milk nutrients [4]. BMB disruption is likely to raise the incidence of mammary disease [5]. It has been found, through research, that the occurrence of mastitis will destroy the integrity of tight junctions (TJs), including a decrease in the expression of TJ proteins (claudin-1, occludin, ZO-1, etc.), thereby increasing their permeability and damaging the health of the mammary gland [6,7].

As a dynamic mechanism of cell self-protection and defense, autophagy functions as a highly efficient approach for removing toxic and harmful substances from cells [8]. It has been gradually proven to remain at a low level under normal physiological conditions, but autophagy will be activated promptly in the case of infection, starvation, and hypoxia [9]. In addition, autophagy participates in diversified inflammatory reactions plus barrier repair as a vital mode for sustaining the normal life activities of cells [10,11]. As revealed by research, the scaffold proteins that are tightly linked can be altered by inducing autophagy [12]. Therefore, autophagy has intimate correlations with inflammation and BMB, while the efficacy of autophagy in treating subclinical mastitis or maintaining the BMB has so far been rarely researched.

Quite a number of studies also proved that an excessive accumulation of reactive oxygen species (ROS) is linked to oxidative stress and inflammation exaggeration [13]. The massively accumulated ROS may lead to oxidative damage by attacking the oxidized proteins and DNA, as well as lipids, which is a feature of multiple inflammatory diseases [14]. Moreover, it is argued that regarding the activation of the NLRP3 inflammasome, ROS functions as an essential triggering factor [15]. Constituted by an apoptosis-associated speck-like protein containing a caspase recruitment domain (ASC) plus NLRP3, together with caspase-1, the NLRP3 inflammasome is a multiprotein complex capable of stimulating caspase-1 activation and further facilitating IL-1β maturation and release [15]. Recent research showed that such representative cytokines can promote multiple cells to produce inflammatory responses, with the ability to trigger or exacerbate inflammation in organs or cells, thus promoting disease development [16,17]. Recent research has described the regulatory roles of ROS generation combined with the activation of NLRP3 inflammasome in BMB disruption in bovines [18]. However, the relationship involving NLRP3 inflammasome, autophagy, and ROS in subclinical bovine mastitis is still unknown.

Thanks to the antioxidant and anti-inflammatory performance mainly attributable to ginsenosides, Ginseng has extensive applications in traditional Chinese medicine [19,20,21]. For example, ginsenoside Rg1 (Rg1, C_42_H_72_O_14_), identified as an active ingredient of ginseng (also known as *Panax ginseng*), is a steroidal saponin with the highest activity and content, of which the anti-inflammatory [22], anti-apoptotic [23], antibacterial [24], and antioxidant [21] abilities have been confirmed through scientific surveys. As revealed by several clinical studies in recent years, Rg1 is efficacious in treating a variety of chronic disorders (e.g., metabolic, psychological, neoplastic, neurological, pulmonary, and cardiovascular diseases) [25]. Moreover, Rg1 has been testified to be able to keep the completeness of TJ proteins, thus exerting protective effects on the intestinal [26], blood–brain [27], and alveolar epithelial barriers [28]. Nevertheless, it is still necessary to elaborate the specific mechanism of the Rg1-mediated protective effect on TJ dysfunction in LTA-triggered bovine mammary epithelial cells (bMECs).

Since intervening in the mammary TJs is likely to exacerbate mastitis, this research was intended to examine the cultured bMEC line (MAC-T) for the role of Rg1 in influencing TJ dysfunction triggered by LTA-mediated inflammation, and probe into the potential molecular events. Furthermore, the mouse model of LTA-triggered mastitis was established to verify the association of Rg1-modulated BMB disruption. Therefore, the aim of the study was to examine the role of excessive autophagy induced by oxidative stress in BMB disruption in subclinical chronic mastitis, and to demonstrate the protective effect of Rg1. It was demonstrated that Rg1 alleviated the LTA-induced BMB disruption by binding to PPAR, which is associated with the interference in the ROS/AMPK/m-TOR/autophagy/NLRP3 inflammasome axis.

## 2. Materials and Methods

### 2.1. Acquisition of Samples

Holstein Friesian cows (42 in total) from Gansu province underwent physical examination. Then the milk samples were subjected to California mastitis test (CMT) together with somatic cell count (SCC) to identify the samples obtained from subclinical mastitis cows and healthy ones [29,30]. Healthy cows had a low udder milk SCC (<10^5^ cells/mL) and a negative CMT result on the sampling day, manifesting no clinical signs of mastitis. The cows with subclinical mastitis were detected with continuously rising SCC (>4 × 10^5^ cells/mL for minimally 3 consecutive times), depending on the SCC determined every month, as well as a positive CMT result and no clinical signs of the disease during sample collection. The mammary tissues (1–2 g per quarter, two quarters per cow) were collected through sterile surgery from healthy cows (n = 4) and subclinical mastitis cows (n = 4). Table 1 shows parity, body condition and the clinical criteria of the individual cows examined. Samples were immediately cryopreserved with liquid nitrogen before protein extraction or stored under glutaraldehyde (2.5%) or paraformaldehyde (4%) for histological, immunohistochemical and echocardiographic analyses.

### 2.2. Prediction of Target Genes Related to Rg1 as Well as Their Intersection on Mastitis

Rg1 was examined using TCMGeneDIT, BATMAN-TCM, TCMSP, and TCMID databases for the identification of chemical components. Next, the information on the target genes, besides chemical compounds of Rg1, was retrieved by virtue of PubChem combined with DrugBank. Additionally, the target genes for curing mastitis were searched in GeneCards plus NCBI. The conversion from protein names into gene names (ENSG identifiers) was achieved by means of UniProt and Ensembl. Finally, the intersection between Rg1 and mastitis (R&M) was obtained, in which the genes deemed the possible target genes of Rg1 affecting mastitis were determined.

### 2.3. Molecular Docking

The docking technique was employed to uncover and forecast the fundamental mechanisms as well as interactions between Rg1 and mastitis-associated target proteins. The PubChem and Protein Data Bank were utilized to produce the 3D crystal structures of Rg1 (CID: 441923) and PPARγ (PDBID: 1WM0), respectively.

Before the start of docking experiments, the surplus protein chains, water molecules and ligands were eliminated via the AutoDockTools 1.5.4, provided by Molecular Graphics Laboratory at the Scripps Research Institute, by virtue of hydrogenation. The docking condition between proteins and small molecules was simulated by AutoDock Vina. As for the results, the binding energy (BE) defined as the weighted average of docking score was applied to appraise the positioning of ligands in terms of reliability and accuracy. The ligand performs better when greater negative energy is utilized [31]. Supplementary Table S1 describes the BE of Rg1 and PPARγ proteins. The result visualization was achieved using the PyMOL (version 2.2.0) and LigPlot (version 2.2) software.

### 2.4. Cell Culture and Treatment

Immortalized bMECs [MAC-T, supplier: Cell Resource Center of the Shanghai Institutes for Biological Sciences, CAS (Shanghai, China)] were cultured at 37 °C using complete DMEM (Gibco, Waltham, MA, USA) mixed by fetal bovine serum (FBS, 10%) + streptomycin (100 µg/mL) + penicillin (100 IU/mL) in a humid incubator with 5% CO_2_.

Prior to experiments, the MAC-T cells were subjected to 24 h of processing with Rg1 (5, 10 or 20 μM as the final concentrations, Med Chem Express, Monmouth Junction, NJ, USA) and/or LTA (0.1, 1 or 10 μg/mL as the final concentration to induce inflammation, Sigma Aldrich, Saint Louis, USA) after 12 h culture with serum-free DMEM. For inhibition studies, the cells were incubated with or without 10 μM MCC950 (NLRP3 inhibitor, Med Chem Express, NJ, USA), 50 μM choroquine (CQ, autophagy inhibitor, Med Chem Express), 5 mM acetylcysteine (NAC, ROS scavenger, Med Chem Express), 10 μM T0070907 (PPARγ inhibitor, Med Chem Express), 10 μM Compound C (CC, AMPK inhibitor, Med Chem Express), and 10 μM GW6471 (PPARα inhibitor, Med Chem Express) for 2 h.

### 2.5. Measurement of Autophagic Flux

Based on the manufacturer’s instructions, mCherry-GFP-LC3 reporter plasmid (1 μL/mL) provided by Beyotime (Nanjing, China) was selected for MAC-T transfection, so as to determine the autophagic flux. Thereafter, the cells underwent grouping and processing through the aforementioned methods. Fluorescence microscopy was performed to observe the cell images.

### 2.6. GSH and ROS Assessment

MAC-T cells were tested by the GSH assay kit offered by Beyotime for GSH level as the manufacturer’s protocol. DCFH-DA (Beyotime) was used for total ROS level measurement with reference to the instructions from the manufacturer. MAC-T cells (5000 cells/well in concentration) were plated in a 96-well microplate and processed as indicated. Next, the DCFH-DA (10 μmol/L)-loaded cells were placed for 30 min away from light (37 °C) and gently cleaned 3 times in PBS. The fluorescence microscope (Nikon, Tokyo, Japan), together with a microplate reader (Gemini XPS, Molecular Devices, Gothenburg, Sweden), was employed to detect total ROS for the fluorescence intensities.

### 2.7. Animal Grouping and Mastitis Modeling

In total, 40 CD-1 female mice expected to give birth within 5–7 days were randomized into the following 5 groups: Rg1 group, control group, LTA-induced mastitis group, as well as LTA-induced mastitis groups plus treatment with Rg1 or rosiglitazone (PPARγ agonist, Med Chem Express). LTA was applied to prepare the mouse model of mastitis in all groups other than control and Rg1 groups. From 3 days prior to mastitis induction with LTA, Rg1 (200 mg/kg/day) or rosiglitazone (10 mg/kg/day) was administered orally. The female mice were separated with their pups before the 3 h induction of mastitis. Following anesthesia with pentobarbital, the fourth pair of nipples of the mice was disinfected with 75% alcohol. Then the nipples were cut by 1 mm using sterilized scissors, followed by injection of 0.2 mg/mL LTA (10 μg) by a 32G needle. Finally, all mice were killed when mastitis was successfully induced in 24 h, and mammary tissue samples were acquired for examination.

### 2.8. Western Blotting Assay

After collection and ice-cold PBS washing, ice-cold RIPA lysis buffer supplemented with 1 mM PMSF was added for processing the cells plus tissues. Western blotting assay was conducted as previously described [32]. The primary antibodies against p-AMPK (Ser485) (1:1000 dilution; #2537; Cell Signaling Technology, Danvers, MA, USA), AMPK (Ser485) (1:1000 dilution; #2532; Cell Signaling Technology), p-mTOR (Ser2448) (1:1000 dilution; #5536; Cell Signaling Technology), mTOR (1:1000 dilution; #2983; Cell Signaling Technology), PPARα (1:1000 dilution; bs-3614R; Bioss, Woburn, MA, USA), PPARδ/β (1:1000 dilution; 28053-1-AP; Proteintech, Rosemont, IL, USA), PPARγ (1:1000 dilution; 16643-1-AP; Proteintech), ZO-1 (1:1000 dilution; 21773-1-AP; Proteintech), occludin (1:1000 dilution; 27260-1-AP; Proteintech), claudin-1 (1:1000 dilution; 28674-1-AP; Proteintech), beclin-1 (1:1000 dilution; 11306-1-AP; Proteintech), LC3 (1:1000 dilution; 14600-1-AP; Proteintech), NLRP3 (1:1000 dilution; 19771-1-AP; Proteintech), ASC (1:1000 dilution; 10500-1-AP; Proteintech), caspase-1 (1:1000 dilution; 22915-1-AP; Proteintech), IL-1β (1:1000 dilution; #12703; Cell Signaling Technology), and β-actin (1:3000 dilution; bs-0061R; Bioss) were utilized. The goat anti-rabbit secondary antibody conjugated with HRP (diluted at 1:3000, bs-0295-HRP; Bioss) was added. The bands were examined using ECL solution, and signal quantification was implemented by virtue of ImageJ 1.44p software.

### 2.9. Histological Analysis

The paraffin-embedded bovine and mice mammary tissues were prepared into 4 μm-thick slices for hematoxylin-eosin staining. The dynamic changes were evaluated under an optical microscope (Olympus-DP73, Tokyo, Japan) for histological analysis of the mammary.

### 2.10. Immunofluorescence Staining

Immunofluorescence staining was conducted based on the procedures in our previous report [32]. Specifically, the overnight incubation (4 °C) of mammary tissue sections was executed by virtue of the rabbit polyclonal antibody against ZO-1 (diluted at 1:100; bs-1329R; Bioss), occludin (diluted at 1:100; bs-10011R; Bioss), claudin-1 (diluted at 1:100; bs-21509R; Bioss) or LC3 (diluted at 1:100; 14600-1-AP; Proteintech). Later, the tissue sections, washed in PBS 3 times, were incubated with the FITC-coupled goat anti-rabbit IgG (H+L) antibody (dilution rate of 1:200; TransGen, Beijing, China) for 45 min. Finally, the cell nuclei received DAPI staining based on the previously described methods, followed by observation and photography of the sections with an Olympus-DP73 optical microscope (Tokyo, Japan).

### 2.11. Assessment of Oxidative Stress in Mammary Glands

The level of lipid peroxidation marker MDA, in addition to the activity of SOD and CAT as enzymatic antioxidants, was examined to appraise the oxidative stress in bovine and murine mammary glands in accordance with the instructions of commercially available kits for SOD (A001-1), MDA (A003-1), plus CAT (A007-1), manufactured by Jiancheng Bioengineering Institute (Nanjing, China).

### 2.12. Transmission Electron Microscopy (TEM)

The 2.5% glutaraldehyde-fixed mammary tissues received 1 h of a third fixation (4 °C) in osmium tetroxide (1%), prior to rehydration, embedding, slicing, and uranyl acetate + citrate double-staining. Finally, the ultrastructure observation of the TJs formed by adjacent bMECs plus autophagy monitoring in bMECs were accomplished using a Hitachi H-7500 transmission electron microscope (Hitachi Ltd., Tokyo, Japan).

### 2.13. Statistical Analysis

Data are shown as the mean ± SD, and GraphPad Prism software (GraphPad Software 9, San Diego, USA) was adopted for result analysis. Normal distribution data were analyzed by unpaired Student’s *t*-test for comparisons between two groups or by one-way ANOVA with a Student–Newman–Keuls test for pairwise comparisons between three or more groups. Data were considered statistically significant at *p* values < 0.05.

## 3. Results

### 3.1. Excessive Autophagy and BMB Disruption in Mammary Glands with Subclinical Bovine Mastitis

**Figure 1:**
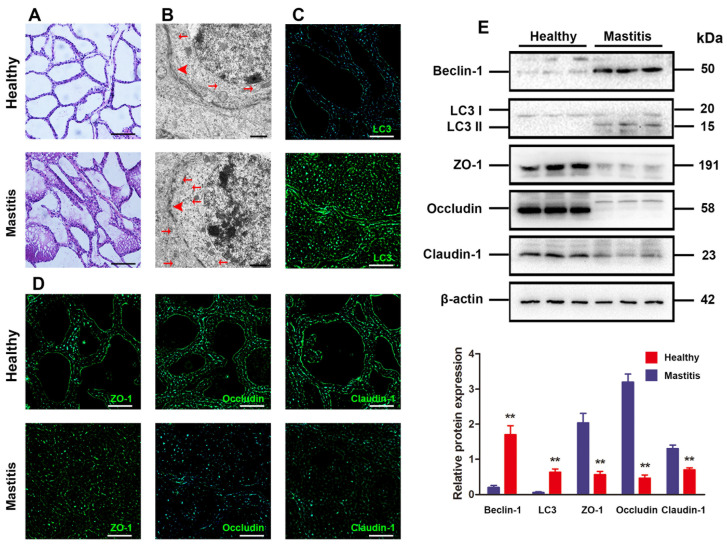
Excessive autophagy and BMB disruption in mammary gland with subclinical bovine mastitis. (**A**) HE staining for histological changes in mammary glands. Original magnification = 200×, scale bar = 100 μm (n = 3). (**B**) Ultrastructure of autophagy and TJs in bMECs observed by TEM (n = 3). Original magnification = 10,000×, scale bar = 500 nm. The red arrowheads indicated TJs, the red arrows indicated autophagic structures. (**C**) Typical immunofluorescence images for localization of LC3 (green) in the mammary glands. Original magnification = 200×, scale bar = 100 μm (n = 3). (**D**) Typical immunofluorescence images for localization of claudin-1, ZO-1, and occludin (green) in the mammary glands. Original magnification = 200×, scale bar = 100 μm (n = 3). (**E**) The relative protein expressions of claudin-1, beclin-1, occludin, ZO-1 and LC3 in mammary glands detected by Western blotting (n = 3). The loading control is set as β-actin, and mean ± SD is used for value expression. ** *p* < 0.01 vs. the healthy group.

First, the histological changes in healthy mammary glands and mammary glands with subclinical bovine mastitis were assessed. It is illustrated in Figure 1A that there were no apparent histological or pathological changes in healthy bovine mammary glandst, while hyperemic edema was observed in the acinar cavity, which was infiltrated with massive inflammatory cells in the mammary glands with subclinical bovine mastitis. Given the pivotal function of BMB formed by adjacent mammary epithelial cells for lactation genesis, its ultrastructural changes in the mammary epithelium were observed under the TEM. The sections made from healthy bovine mammary glands displayed clear and integrated TJ ultrastructure formed by adjacent mammary epithelial cells, while the TJ ultrastructure was disassembled in the mammary glands with subclinical mastitis (Figure 1B).

There is increasing evidence that BMB function has an intimate correlation with autophagy. On this basis, the autophagy change in the mammary glands with subclinical mastitis and in healthy mammary glands was also observed. As for the mammary glands with subclinical mastitis, the number of autophagic vesicles in the cytoplasm of mammary epithelium cells dropped distinctly, with visible swollen endoplasmic reticulum, vacuoles, and disorderly arranged mitochondria (Figure 1B). Subsequently, the mammary glands with subclinical mastitis and healthy mammary glands were also examined for concentrations of TJ-related proteins and the expression of autophagy markers (Figure 1C,D). Tissue immunofluorescence analysis showed that when compared with the healthy mammary gland, the mammary glands with subclinical mastitis exhibited notably lowered ZO-1, occludin and claudin-1 expressions, as well as clearly elevated LC3 plus beclin-1 expressions at the protein level (Figure 1B,C). Moreover, Western blotting assay also manifested remarkably decreased ZO-1, claudin-1 and occludin expressions in subclinical mastitis by contrast with those in the healthy ones, while the opposite trends in beclin-1 and LC3 expressions were obtained (Figure 1E). Overall, the aforementioned findings suggest the possible relation of excessive autophagy to BMB disruption in mammary glands with subclinical bovine mastitis.

### 3.2. Rg1 Inhibited LTA-Induced Excessive Autophagy to Alleviate TJ Dysfunction

**Figure 2:**
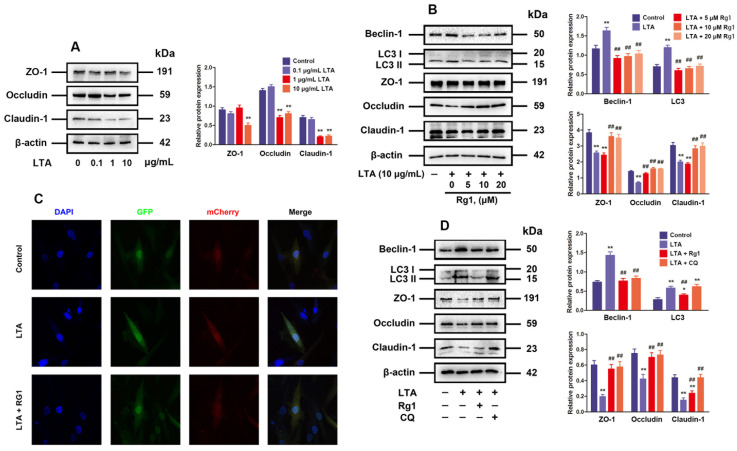
Rg1 inhibited LTA-induced excessive autophagy to alleviate TJ dysfunction. (**A**) MAC-T cells processed for 24 h by LTA at 0.1, 1 and 10 μg/mL. Western blotting for proteins associated with TJs (ZO-1, occludin, and claudin-1). (**B**) MAC-T cells undergoing 24 h LTA (10 μg/mL) and/or Rg1 (at required concentrations) treatment. Western blotting for proteins in relation to autophagy and TJs. (**C**) MAC-T cells subjected to 24 h of LTA (10 μg/mL) and/or Rg1 (20 μM) processing. After the transfection of MAC-T cells with adenovirus plus autolysosome quantitation via mCherry-GFP-LC3, autophagy was visually observable. Original magnification = 400×. (**D**) MAC-T cells under 24 h of treatment by LTA (10 μg/mL), Rg1 (20 μM), and/or CQ (50 μM). Western blotting for proteins correlated with autophagy and TJs. The mean ± SD is used for value presentation; compared with the control group, * *p* < 0.05, ** *p* < 0.01; compared to the LTA treatment group, ## *p* < 0.01.

For the study of subclinical bovine mastitis, LTA stimulation is a well-established method [33]. As shown in Figure 2A, when MAC-T cells were exposed to 10 μg/mL LTA stimulation, occludin, claudin-1 and ZO-1 declined obviously at the expression level. In addition, Rg1 treatment dose-dependently upregulated these TJ-related protein expressions while downregulating Beclin-1 and LC3 expressions in LTA-induced MAC-T cells (Figure 2B). Furthermore, it was also found through mCherry-GFP-LC3 fluorescence analysis that the formation of autophagosome and autolysosome punctum was increased in MAC-T cells exposed to LTA by comparison to those in the control group, which was effectively inhibited by 20 μM Rg1 treatment under LTA induction (Figure 2C).

CQ was applied to explore the correlation of TJs with autophagy in this study. As indicated by Western blotting, CQ showed a similar effect to Rg1, which not only abolished the inhibition effect of LTA on TJ-associated protein expression, but also reduced the expression of beclin-1 and LC3 (Figure 2D). To sum up, the inhibition of excessive autophagy is related to Rg1-induced recovery of MAC-T cell TJ dysfunction

### 3.3. Rg1 Inhibited LTA-Induced TJ Dysfunction via Blocking the ROS/Autophagy/NLRP3 Inflammasome Axis

**Figure 3:**
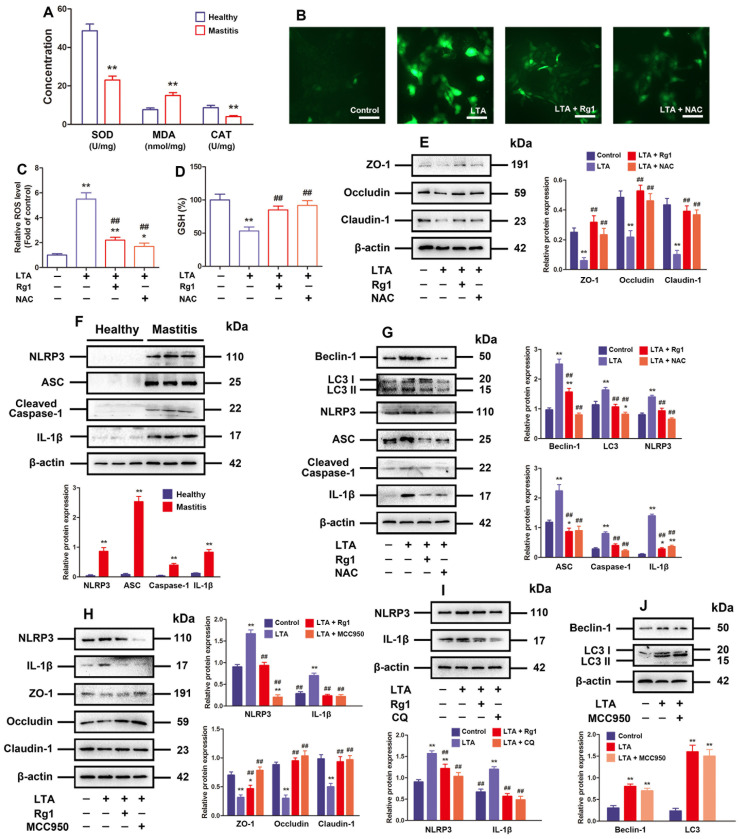
Rg1 inhibited LTA-induced TJ dysfunction by blocking the ROS/autophagy/NLRP3 inflammasome axis. (**A**) SOD, MDA, and CAT content in subclinical bovine mastitis and healthy mammary tissues detected (n = 4). MAC-T cells that had undergone treatment by LTA (10 μg/mL), Rg1 (20 μM), and/or NAC (5 mM) for 24 h. (**B**) DCFH-DA probe for ROS level evaluation in MAC-T cells (original magnification = 400×, scale bar = 50 μm.). (**C**) Quantification of MAC-T cells for ROS content (n = 6). (**D**) Calculated GSH level in MAC-T cells (n = 6). (**E**) Western blotting for ZO-1, occludin, and claudin-1 (proteins related to TJs). (**F**) Relative protein expressions concerning ASC, IL-1β, NLRP3, and caspase-1 in mammary glands determined by means of Western blotting. (**G**) Western blotting for the TJ-related and NLRP3 inflammasome-related proteins. (**H**) MAC-T cells subjected to LTA (10 μg/mL), Rg1 (20 μM), and/or MCC950 (10 μM) processing for 24 h. Western blotting for the NLRP3 inflammasome-related and TJ-related proteins. (**I**) MAC-T cells under LTA (10 μg/mL), Rg1 (20 μM), and/or CQ (50 μM) treatment for 24 h. Western blotting for NLRP3 and IL-1β proteins. (**J**) Twenty-four hour treatment for MAC-T cells using LTA (10 μg/mL), and/or MCC950 (10 μM). Western blotting for beclin-1 and LC3 proteins. The mean ± SD is the expression format for values; compared with the control group, * *p* < 0.05, ** *p* < 0.01; compared to the LTA treatment group, ## *p* < 0.01.

Inflammation, generally concomitant with an increment in ROS and oxidative stress within cells, is well known to be able to induce autophagy [34]. According to Figure 3A, the mammary gland with subclinical bovine mastitis clearly exhibited lower CAT and SOD content and prominently higher MDA content than the healthy ones, indicating that the mammary gland with subclinical bovine mastitis was undergoing oxidative stress. Furthermore, the results of in vitro experiment revealed notably lowered GSH but elevated ROS levels when MAC-T cells were exposed to LTA in contrast to those in the control group, but Rg1 and NAC processing was able to effectively reduce ROS and increase GSH levels in MAC-T cells under LTA induction (Figure 3B–D). In addition, Western blotting results also presented that administration of Rg1 or NAC abolished the inhibitive effect of LTA on TJ-related protein expression, and repressed beclin-1 and LC3 at the expression level (Figure 3E,G).

ROS possesses the crucial function of triggering NLRP3 inflammasome activation, and the latter can cause barrier dysfunction as well [35]. According to Western blotting results, the mammary gland with subclinical bovine mastitis exhibited obviously increased protein expressions concerning ASC, cleaved-caspase-1, NLRP3, and IL-1β compared with the healthy ones (Figure 3F). Furthermore, our in vitro experiment revealed that the administration of MCC950 resembled Rg1 in terms of the effect of abolishing the inhibitory effect of LTA on TJ-related protein expression (Figure 3H).

The roles of CQ and MCC950 in influencing the expression of autophagy-related and NLRP3 inflammasome-related proteins were separately explored, so as to elucidate the correlation of NLRP3 inflammasome with autophagy. Our results showed that CQ significantly inhibited NLRP3 and IL-1β protein expressions, while MCC950 had no significant effect on beclin-1 and LC3 protein expression (Figure 3I,J). Taken together, Rg1 can recover the LTA-induced TJ dysfunction by inhibiting the ROS/autophagy/NLRP3 inflammasome axis.

### 3.4. Rg1 Regulated the ROS/AMPK/mTOR Signaling Pathway to Inhibit LTA-Induced Excessive Autophagy

**Figure 4:**
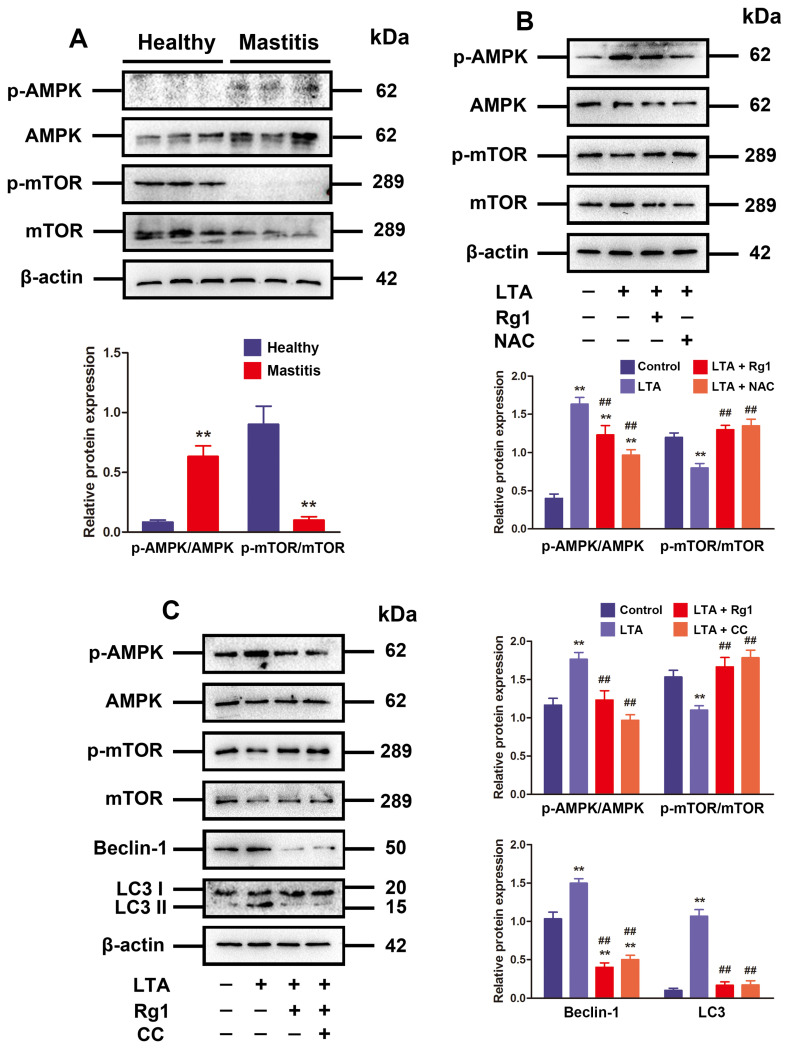
Rg1 inhibited LTA-induced excessive autophagy by regulating the ROS/AMPK/mTOR signaling pathway. (**A**) Relative protein expressions of mTOR, AMPK, p-AMPK, and p-mTOR in mammary glands determined by means of Western blotting (n = 3). (**B**) Rg1 (20 μM), LTA (10 μg/mL), and/or NAC (5 mM) added to treat MAC-T cells for 24 h. Western blotting for AMPK, mTOR, p-AMPK, and p-mTOR protein. (**C**) MAC-T cells subjected to 24 h of LTA (10 μg/mL), Rg1 (20 μM), and/or CC (10 μM) treatment. Protein expressions of AMPK, mTOR, beclin-1, p-mTOR, LC3, and p-AMPK detected by Western blotting. The mean ± SD is adopted to describe values; compared to the control group, ** *p* < 0.01; compared to the LTA treatment group, ## *p* < 0.01.

Since the AMPK/mTOR signaling pathway is known for its role in inducing autophagy, the p-mTOR plus p-AMPK content in bovine mammary gland was examined first [36]. According to the Western blotting results in Figure 4A, the mammary glands with subclinical bovine mastitis exhibited obviously increased p-AMPK expression and reduced p-mTOR expression compared with the healthy ones. The implication of AMPK in triggering autophagy to respond to ROS-induced stress has been strongly evidenced by a number of recent reports [37,38]. Our in vitro results showed that the stimulation of LTA on the expression of p-AMPK and the inhibition effect on the p-mTOR expression in MAC-T cells were eliminated by Rg1 or CQ administration (Figure 4B). Next, the administered CQ alleviated the promoting effect on beclin-1 and LC3 expressions in MAC-T cells induced by LTA (Figure 4C). It can be concluded that Rg1 modulates oxidative stress to inhibit excessively activated autophagy, thus influencing the downstream AMPK/ mTOR signaling pathways.

### 3.5. Rg1 Inhibited LTA-Induced Excessive Autophagy and Recover TJ Dysfunction by Virtue of Oxidative Stress Suppression Plus PPARγ Upregulation

**Figure 5:**
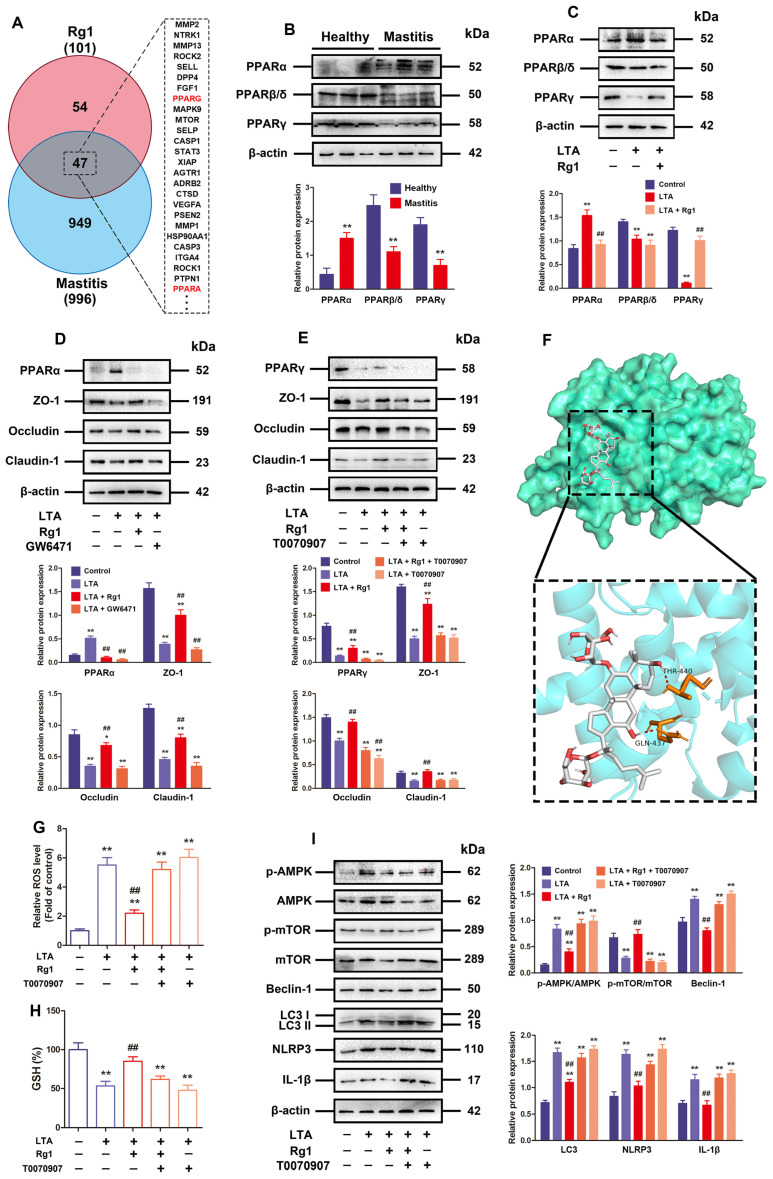
Repressed oxidative stress plus raised PPARγ are necessary for Rg1 to inhibit LTA-induced excessive autophagy and TJ recovery. (**A**) Number of intersecting NAFLD, mastitis and R&M target genes. (**B**) Relative expressions of PPARα, PPARβ/δ, and PPARγ proteins in mammary glands determined by means of Western blotting (n = 3). (**C**) MAC-T cells subjected to 24 h of LTA (10 μg/mL) and/or Rg1 (20 μM) treatment. Western blotting for PPARα, PPARβ/δ, and PPARγ protein. (**D**) MAC-T cells undergoing 24 h disposal by LTA (10 μg/mL), Rg1 (20 μM), and/or GW6471 (10 μM). Western blotting for PPARα and TJ-related proteins. (**E**) MAC-T cells under 24 h of treatment using LTA (10 μg/mL), Rg1 (20 μM), and/or T0070907 (10 μM). Western blotting for PPARγ and TJ-related proteins. (**F**) Docking of PPARγ with Rg1. Rg1 interacts with 2 amino acids, THR 440 and GLN 437, adjacent to the PPARγ active site, forming crucial binding forces in the periphery of the active site. (**G**) ROS level quantification in MAC-T cells (n = 6). (**H**) GSH level in MAC-T cells was calculated (n = 6). (**I**) Western blotting for autophagy-related plus NLRP3 inflammasome-related proteins like AMPK, mTOR, p-AMPK, and p-mTOR. The mean ± SD is used to express values; compared with the control group, * *p* < 0.05, ** *p* < 0.01; compared with the LTA treatment group, ## *p* < 0.01.

Next, how Rg1 affects the ROS/autophagy/NLRP3 inflammasome axis was studied. As shown in Figure 5A, after removing the repeated names, 101 corresponding Rg1 target genes were found (Supplementary Table S2). In total, 996 target genes in relation to mastitis were screened out (Supplementary Table S3), covering 47 R&M target genes (Supplementary Table S4, contributing to 46.53% of Rg1 and 4.72% of mastitis) by using the Draw Venn Diagram tool. Among them, PPARα and PPARγ were found.

Peroxisome proliferator-activated receptors (PPARs) refer to a class of adhesive-activated nuclear receptors exerting an effect on the transcriptional modulation of energy balance, oxidative stress, inflammation, and lipid metabolism [38]. There have been three clarified subtypes of PPARs: PPARα, PPARδ/β, and PPARγ. Based on Western blotting in the present research, the mammary glands with subclinical bovine mastitis exhibited obviously increased PPARα expressions but reduced PPARδ/β and PPARγ expressions, in contrast to the healthy mammary glands (Figure 5B). It was discovered by in vitro survey that LTA administration significantly increased PPARα expressions and reduced PPARδ/β and PPARγ expression, which were consistent with the results from bovine mammary glands, while Rg1 treatment recovered PPARγ, inhibited PPARα expression, and had no significant effect on PPARδ/β expression (Figure 5C). Subsequently, the PPARα inhibitor GW6471 and PPARγ inhibitor T0070907 were further applied to explore the correlation of PPARα or PPARγ with TJ dysfunction in LTA-induced MAC-T cells. Interestingly, the inhibition of PPARα had no significant effect on TJ-related protein expression in LTA-induced MAC-T cells, but the inhibition of PPARγ blocked the recovery effect of Rg1 on TJ-related protein expression (Figure 5D,E). Thus, PPARγ became the focus of in-depth investigations. Molecular docking results showed that Rg1 possessed certain affinity to the crystal structure of the PPARγ protein (Figure 5F). In addition, the inhibition of PPARγ by T0070907 prominently mitigated the inhibitory role of Rg1 in LTA-induced MAC-T cells’ ROS production and increased levels of GSH (Figure 5G,H). Moreover, the LTA + Rg1 + T0070907 group displayed higher expression levels of p-AMPK, beclin-1, LC3, NLRP3, IL-1β and lower p-mTOR, ZO-1, occludin and claudin-1 expressions than the LTA plus Rg1 group (Figure 5I). In summary, PPARγ upregulation is a necessary factor for Rg1 during interference in the ROS/autophagy/NLRP3 axis as well as in the subsequent recovery of TJ dysfunction.

### 3.6. Rg1 Alleviated BMB Disruption by Modulating Autophagy Signaling In Vivo

**Figure 6:**
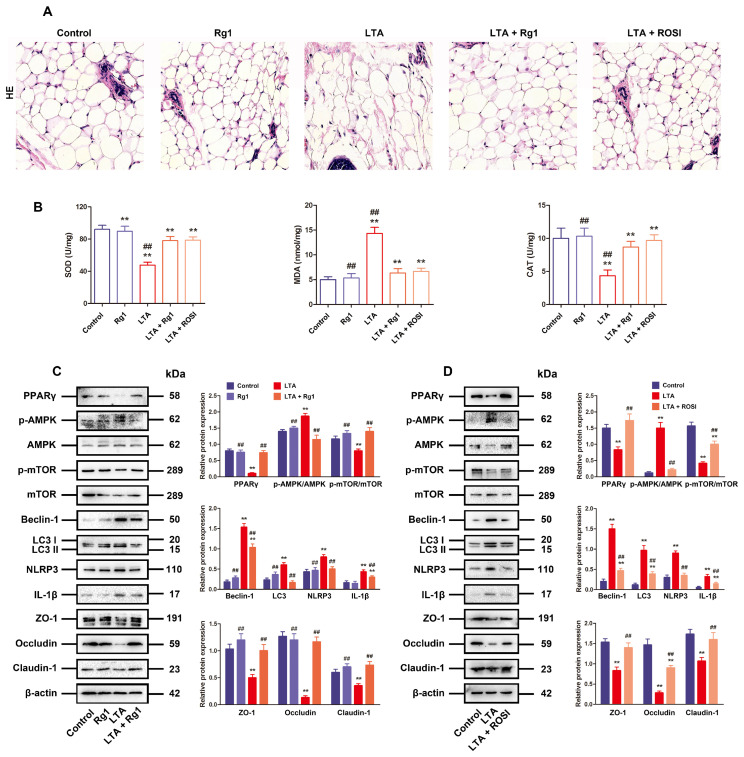
Rg1 alleviated BMB disruption through regulating autophagy signaling in organisms. Five groups were set up for the mice: control group (no treatment with LTA), Rg1 (no LTA, 200 mg/kg) group, model group (LTA injection, no treatment), Rg1 (200 mg/kg) + LTA treatment group, and rosiglitazone (10 mg/kg) + LTA treatment group. (**A**) Hematoxylin-eosin (HE) staining for general examination of mammary glands (original magnification = 200×, scale bar = 100 μm), together with immunofluorescence images for LC3, ZO-1, occludin and claudin-1 (original magnification = 400×, scale bar = 50 μm). (**B**) Examined variations in SOD, MDA, and CAT content. (**C**,**D**) Western blotting for determining the relative protein expressions of AMPK, PPARγ, p-AMPK, mTOR, p-mTOR, autophagy-related, NLRP3 inflammasome-related and TJ-related proteins. The mean ± SD as the value format and β-actin as the loading control. Compared with the control group, ** *p* < 0.01; Compared with the LTA treatment group, and ## *p* < 0.01.

For the purpose of clarifying whether Rg1 recovers TJs to alleviate BMB disruption by activating PPARγ while restraining oxidative stress in organisms, a typical mouse model of mastitis induced by LTA was established. As expected, pathological examination by HE staining showed that in comparison to that in the control and Rg1-alone group, LTA treatment led to typical damage to the mammary gland in the model group. Furthermore, the administration of Rg1 or PPARγ agonist rosiglitazone significantly improved LTA-induced mammary injury (Figure 6A). Furthermore, when compared with the model group, treatment with Rg1 or rosiglitazone significantly decreased the levels of MDA, while increasing SOD and CAT in LTA-induced mastitis mice (Figure 6B). Lastly, it was uncovered via Western blotting that Rg1 or rosiglitazone could lower the levels of p-AMPK, NLRP3, beclin-1, LC3, and IL-1β, while restoring the levels of claudin-1, PPARγ, occludin, ZO-1, and p-mTOR, in mousse mastitis, in agreement with the results obtained in LTA-treated MAC-T cells (Figure 6C,D). To sum up, Rg1 alleviates TJ dysfunction by binding to PPARγ, which is associated with the interference in the ROS/AMPK/mTOR/autophagy/NLRP3 axis in LTA-caused mastitis, contributing to the recovery of BMB function.

## 4. Discussion

Mastitis refers to an inflammatory reaction which occurs in the bovine mammary gland, with a direct association with reduced milk yield and altered milk quality, leading to substantial economic losses to dairy farms and the dairy industry. There is a direct relationship between the mastitis-induced reduction in milk yield and bMECs dysfunction, including the impaired cell integrity, weakened cell viability and enhanced apoptosis [39]. Mammary epithelial cells form the BMB through special connecting structures, such as TJs. However, when mastitis occurs, excessive inflammatory cytokines may result in a serious pro-inflammatory response that destroys the TJs’ integrity, thereby leading to the exacerbation of tissue injury and destroying the secretory function of bMECs [4,5]. The present study demonstrated that Rg1 might recover the TJs in LTA-induced inflammation in MAC-T cells and the BMB function in a mastitic mouse model. The underlying mechanism revealed that the protective role of Rg1 could protect TJ function in MAC-T cells by inhibiting the AMPK/mTOR/autophagy/NLRP3 signaling pathway, in part by stimulating the reduction in PPARγ-activated ROS generation.

As a cell decomposition mode, autophagy is correlated with the transportation of materials from cytoplasm to lysosomes to some extent [8]. It has been reported to participate in diversified diseases and function as a crucial mechanism of cell survival, thus affecting the intestinal barrier function [10,11]. Nevertheless, uncontrolled or overstimulated autophagy may arouse the pathological conditions and finally trigger autophagic cell death [40]. Reports have proved that lipopolysaccharide (LPS)-induced inflammation in cultured intestinal epithelial cells can serve as an autophagy stimulator to induce TJ dysfunction [34]. It was demonstrated by this research that the autophagy level was elevated in tissues with mastitis and the integrity of the TJs was broken compared with those in healthy ones. LTA, as a cell wall component obtained from *S. aureus*, can stimulate chronic and subclinical mastitis in bovines [2,3]. Our data also demonstrated that the LTA induced inflammation could increase the autophagy level and lead to TJ dysfunction in the mouse model, together with cell experiments. Taken together, our data corroborated that the overstimulated autophagy induced by LTA probably serves as a pivotal factor for BMB disruption in subclinical bovine mastitis.

It was demonstrated that Rg1 has a curing effect on inflammation-induced damage due to its anti-inflammatory and antioxidant properties [26,27,28]. The role of Rg1 in influencing TJ dysfunction in MAC-T cells, along with its relation to the relief of mastitis-induced injury, was analyzed in this research. It was found that Rg1 markedly restored the TJs in LTA-induced MAC-T cells and BMB in the mouse mastitis model, and significantly reduced the autophagy level. Moreover, the lysosomal inhibitor CQ was used to determine the correlations between Rg1, TJs, and autophagy in this study. As expected, our data demonstrated that Rg1 attenuated LTA-induced TJ dysfunction in MAC-T cells by inhibiting autophagy.

Although the ability of Rg1 to suppress MAC T cell autophagy to alleviate TJ dysfunction progression has been clarified, there are still two problems. First, how does autophagy affect TJ dysfunction? Second, how does Rg1 affect autophagy? According to previous studies, ROS-induced excessive autophagy caused myocardial damage after myocardial infarction [37]. It was well known that mastitis-induced excessive ROS not only destroys the BMB but also injures the mammary glands of dairy cows, thus decreasing milk production and milk quality [18,39]. Our study showed that the ROS level was significantly increased in subclinical mastitis tissues compared with that in healthy ones. Moreover, after LTA stimulation, the intracellular ROS level was also increased, as indicated by in vivo and in vitro studies, implying that ROS has an impact on mastitis. In addition, similar to Rg1, ROS inhibitor NAC can effectively reduce the expression of autophagic proteins while increasing TJ-related protein expression. Taken together, our findings showed that Rg1 can inhibit ROS-induced excessive autophagy to improve the TJs in MAC-T cells. Apart from autophagy, ROS could also trigger the NLRP3 inflammasome. Our previous study demonstrated that Rg1 mitigated the LPS-triggered TJ disruption in porcine intestines by inhibiting the NLRP3 inflammasome [41]. NLRP3 inflammasome activation in subclinical mastitis and after LTA stimulation in MAC-T cells and murine mammary glands was confirmed in the present study by the upregulation of NLRP3, ASC, cleaved-caspase-1 and IL-1β. Both Rg1 and NLRP3 inhibitor MCC950 can effectively reduce NLRP3 inflammasome-associated proteins from the aspect of expression to improve TJs in LTA-induced MAC-T cells. Previous studies have demonstrated the crucial function of autophagy in the removal of misfolded proteins and ATP, as well as proinflammatory cytokines capable of activating the NLRP3 inflammasome [42]. Additionally, it has been gradually evidenced that autophagy-related proteins can package and degrade ASC and NLRP3, the components of the NLRP3 inflammasome [43]. Unfortunately, the modulating effect of autophagy on the NLRP3 inflammasome in subclinical bovine mastitis is still elusive. For the purpose of exploiting the relationship of the NLRP3 inflammasome with autophagy, an in vitro model was constructed to test the effect of CQ and MCC950 on autophagy-related and NLRP3 inflammasome-related protein expression, respectively, which sufficiently testified the upstream role of excessive autophagy in the NLRP3 inflammasome to induce the procession of TJ dysfunction [36,37]. Furthermore, AMPK and mTOR have been recognized as crucial pathways involved in ROS-induced excessive autophagy and subsequent cell impair. Being a sensor of energy molecules, AMPK is able to facilitate autophagy by downregulating mTOR phosphorylation [44]. In the meantime, mTOR acts as a critical serine/threonine kinase for cellular metabolism as well as a regulatory target for autophagy [45]. As shown in this research, Rg1 attenuated AMPK phosphorylation but enhanced mTOR phosphorylation in vivo and in vitro, and the CC-induced AMPK inhibition exerts a similar therapeutic effect to Rg1 on LTA-induced MAC-T cells. To sum up, RG1 can recover TJs by inhibiting the ROS/AMPK/autophagy/NLRP3 inflammasome axis.

With the confirmed action mechanism plus its effectiveness, computational bioinformatics was applied to further evaluate the BMB protecting effect of Rg1; PPARγ participates in ROS modulation by virtue of the role of a nuclear receptor transcription factor by controlling redox along with biosynthetic procedures in mitochondria [46]. Regarding the three isotypes of PPAR, PPARγ has high expression in bovine white adipose tissue, and it is also relatively abundantly expressed in mammary tissues [47]. In addition to the regulatory role in milk fat synthesis, PPARγ also functions positively in response to mammary infection in the host [48]. Moyes et al. found that intramammary infusion of dairy cows with Strep. uberis resulted in a concurrent reduction in PPAR pathway downregulation as well as milk fat synthesis [49]. In addition, many studies also demonstrated the potential role of Rg1 as a PPARγ ligand, which suppresses the cerebral ischemia-reperfusion-induced neuron injury by activating PPARγ [50,51]. In this study, the results of network pharmacology and molecular docking assays confirmed that Rg1 acted as a potential ligand of PPARγ and restored the expression of PPARγ in vivo and in vitro. Furthermore, its activation could lower the degree of oxidative stress. Subsequently, T0070907 was employed to repress PPARγ expression to further validate the experimental results, and it was found that the therapeutic effect of Rg1 on TJs and its inhibitory effect on the ROS/AMPK/autophagy/NLRP3 inflammasome axis were abolished by T0070907. Finally, the PPARγ agonist rosiglitazone was utilized in an in vitro study to confirm the results mentioned above, in line with previous findings.

## 5. Conclusions

**Figure 7:**
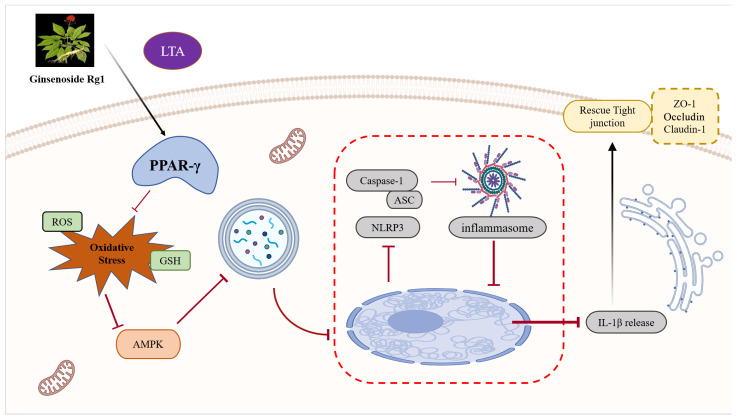
Proposed mechanism of Rg1 in alleviating BMB disruption in cases of subclinical bovine mastitis by regulating oxidative stress-induced excessive autophagy.

In conclusion, our findings confirm that Rg1 efficiently ameliorates LTA-triggered BMB disruption in mice while restraining the TJ dysfunction in MAC-T cells. It is probably attributable to the inhibitory effect exerted by Rg1 on oxidative stress and excessive autophagy in the case of mastitis (Figure 7). As a result, the present research data further support the mammary protecting effect and mechanism of Rg1. It is expected that Rg1 can be used as an efficacious drug for treating mastitis and to lay effective foundations as well as guidelines for clinical treatment.

## Figures and Tables

**Table 1:** Results of clinical examination.

Cow ID	T (°C)	Parity	BCS *	SCC (Cells/mL)
Healthy cow 1	38.2	2	4	5 × 10^4^
Healthy cow 2	38.4	3	4.25	1 × 10^4^
Healthy cow 3	38.4	4	4	9.8 × 10^4^
Healthy cow 4	38.3	3	4.25	2 × 10^4^
Subclinical mastitis cow 5	39.7	5	3.25	2.31 × 10^6^
Subclinical mastitis cow 6	39.6	2	3.5	4.72 × 10^6^
Subclinical mastitis cow 7	39.4	2	3.5	3.12 × 10^6^
Subclinical mastitis cow 8	39.2	3	3.75	8.6 × 10^5^

* Body condition score on a scale of 1–5.

## Data Availability

All of the data are contained within the article.

## Supplementary Materials

The following supporting information can be downloaded at: https://www.mdpi.com/article/10.3390/antiox13121446/s1, Table S1. Pharmacological and molecular properties of Ginsenoside Rg1. Table S2. 101 Rg1 related targets. Table S3. 996 Mastitis related genes. Table S4. 47 Rg1 & Mastitis target genes.

## Abbreviations

ASC	Caspase recruitment domain
BMB	Blood–milk barrier
bMECs	Bovine mammary epithelial cells
Cat	Catalase
CC	Compound C
CMT	California mastitis test
CQ	Chloroquine
DCFH	Dichloro-dihydro fluorescein
HE	Hematoxylin-eosin staining
LPS	Lipopolysaccharide
LTA	Lipoteichoic acid
MDA	Malondialdehyde
NAC	Acetylcysteine
Rg1	Ginsenoside Rg1
ROS	Reactive oxygen species
SCC	Somatic cell count
SOD	Superoxide dismutase
TEM	Transmission electron microscopy
TJs	Tight junctions
