# Supplementary material for: Ginsenoside Rg1 Alleviates Blood–Milk Barrier Disruption in Subclinical Bovine Mastitis by Regulating Oxidative Stress-Induced Excessive Autophagy
Source: Antioxidants (Basel). 2024 Nov 24;13(12):1446. doi: 10.3390/antiox13121446 (PMC11673002; doi:10.3390/antiox13121446)
Supplement: Supplementary file 1 [file antioxidants-13-01446-s001.zip › Supplementary Table S1.pdf]

S1 Table. Pharmacological and molecular properties of Ginsenoside Rg1.

MW	AlogP	Hdon	Hacc	OB (%)	Caco-2	BBB	DL	FASA	TPSA	RBN
801.01	1.13	10	14	10.04	-2.27	-3.50	0.28	0.24	239.22	10

Abbreviations: Caco-2, Caco-2 permeability; OB, oral bioavailability; DL, drug likeness; BBB, blood–brain barrier.
